# Wearable Fitness Trackers to Predict Clinical Deterioration in Maintenance Hemodialysis: A Prospective Cohort Feasibility Study

**DOI:** 10.1016/j.xkme.2021.04.013

**Published:** 2021-06-29

**Authors:** Meaghan Lunney, Natasha Wiebe, Elizabeth Kusi-Appiah, Alexander Tonelli, Rachel Lewis, Reed Ferber, Marcello Tonelli, Alberta Kidney Disease Network

**Affiliations:** 1 Department of Community Health Sciences, Cumming School of Medicine, University of Calgary, Calgary, AB, Canada; 2 Department of Nephrology, Cumming School of Medicine, University of Calgary, Calgary, AB, Canada; 3 Department of Medicine, Cumming School of Medicine, University of Calgary, Calgary, AB, Canada; 4 Faculty of Kinesiology, University of Calgary, Calgary, AB, Canada; 5 Department of Medicine, University of Alberta, Edmonton, AB, Canada; 6 École Fédérale Polytechnique de Lausanne, Lausanne, Vaud, Switzerland

**Keywords:** Fitness tracker, hemodialysis, prediction, digital health

## Abstract

**Rationale & Objective:**

People receiving hemodialysis often require urgent care or hospitalizations. It is possible that reductions in a patient’s level of physical activity may serve as an “early warning” of clinical deterioration, allowing timely clinical intervention. We explored whether step count could serve as a trigger for deterioration.

**Study Design:**

Prospective observational cohort feasibility study.

**Setting & Participants:**

We recruited consenting adult participants from outpatient dialysis clinics in Calgary, AB, between June 28, 2019, and October 10, 2019.

**Exposure and Outcomes:**

Participants wore a wristband fitness tracker for 4 weeks. Activity data from the trackers were imported weekly into the study database. Demographic, clinical management, functional impairment, and frailty were assessed at baseline. Clinical events (urgent care and emergency department visits and hospitalizations) were monitored during the observation period.

**Analytical Approach:**

Box and whisker plots and line plots were used to describe each participant’s daily steps. Adjusted rate ratios (and 95 % confidence intervals) were calculated to assess the associations between the number of steps taken each day and potential predictors.

**Results:**

Data from 46 patients were included; their median age was 64 years (range, 22 to 85), and 63 % were men. The median number of steps taken per day was 3,133 (range, 248-13,753). Fourteen events among 11 patients were reported. Within patients, step count varied considerably; it was not possible to identify a patient-specific normal range for daily step count. There was no association between step count and the occurrence of clinical events, although the number of events was very small.

**Limitations:**

The number of participants was relatively small, and there were too few events to model to examine whether step count could predict clinical deterioration.

**Conclusions:**

Within-patient variation in daily step count was too high to generate a normal range for patients. However, patient-specific norms over a longer period (3- or 7-day periods) appear potentially worthy of future study in a larger sample and/or over a longer follow-up.

## Graphical abstract

**Figure:**
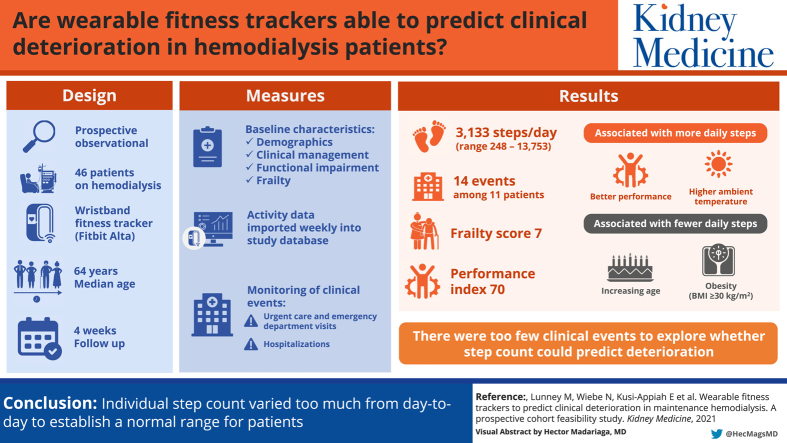

**Plain-Language Summary**

Physical activity may be a useful measure to indicate health status. Our study explored the potential of using a commercial fitness tracker to predict clinical deterioration among people with kidney failure receiving maintenance hemodialysis. We observed 46 patients for 4 weeks to monitor their daily step count and any adverse events (e.g., hospitalizations). The step count was too inconsistent within each person to identify a normal range, making it difficult to use step count as a predictive indicator. Future work in this area to consider other ways of monitoring step count (for example, a rolling average) over a longer follow-up period with more events may help explore the potential of using fitness trackers to predict deterioration.

Unplanned hospitalizations are frequent in people receiving maintenance hemodialysis. Data from the United States Renal Data System program indicate that readmission rates among hemodialysis patients are higher compared to those with other serious chronic diseases^1^ and reducing readmission is an important quality indicator for dialysis care.^2^ The leading causes of hospitalization among hemodialysis patients include extracellular fluid volume overload, infection, and progressive frailty/weakness. Some hospitalizations likely result from clinical situations evolving over minutes to hours. Others likely progress more slowly (over days to weeks), such as when a patient becomes progressively volume overloaded, or contracts a relatively indolent but progressive infection. It is possible but untested that variations in a patient’s physical activity may serve as an early warning of such progressive deterioration, triggering a detailed clinical assessment followed by timely intervention and treatment.

A prerequisite for using a reduction in physical activity as a trigger for clinical assessment would be a more comprehensive understanding of how physical activity varies in and between maintenance hemodialysis patients. For example, it is not known whether patient-specific or age-specific norms could be best used for this purpose. Existing data on objective measures of physical activity in hemodialysis patients are limited, with no studies exploring within-patient patterns identified.

We designed this study to systematically collect the objectively measured physical activity of patients in a prevalent cohort of people receiving maintenance hemodialysis in Alberta using the commercially available Fitbit Alta HR tracker. The Fitbit Alta is a trademark of Fitbit, Inc, and/or its affiliates in the United States and other countries. We were specifically interested in differences in the activity patterns of individual patients (within-patient variability), but also in relation to other patients (between-patient variability). The ultimate goal of the study was to assess the feasibility of using physical activity to identify patients who were deteriorating clinically.

## Methods

This was a prospective observational study of consenting adult patients. The University of Calgary research ethics board approved this study (REB18-2120) and the study was in adherence to the Declaration of Helsinki. This study is reported according to STROBE guidelines.^3^

### Population

We recruited participants from outpatient hemodialysis clinics associated with the Southern Alberta Renal Program in Calgary, Alberta between June 28, 2019, and October 10, 2019. Eligible patients were aged ≥ 18 years and had been established on maintenance hemodialysis treatment for the treatment of kidney failure at an eligible clinic for at least 1 month. Exclusion criteria included self-reported or clinically obvious inability to wear a Fitbit Alta wristband on an upper extremity (e.g., bilateral orthopedic casts, bilateral cellulitis, bilateral amputation), inability to ambulate (e.g., paraplegia, lower limb amputation, usual wheelchair use), or a known history of cutaneous allergy or sensitivity to nickel (present in the Fitbit tracker).

### Study Measures

Participants were fitted with a Fitbit tracker of appropriate size and were asked to wear it at all times except when bathing or cleaning the tracker for 4 weeks. Fitbit wristbands were placed on the contralateral side to the functional arteriovenous access. If no functional arteriovenous access was present, the nondominant arm was used. Based on patient preference, the research assistant either charged the tracker during a weekly follow-up session or lent out the charger for patients to charge the device at their homes.

In addition to a number of demographic and clinical management characteristics at baseline, we assessed the degree of functional impairment using the Karnofsky Performance Index,^4^ and the degree of fitness or frailty using the Rockwood Clinical Frailty Scale.^5^ The Karnofsky Performance Index ranges from 0 to 100, with 100 indicating no impairment and no special care needs. The Rockwood Clinical Frailty Score ranges from 1 to 9, where 1 indicates a very fit individual. Events were defined as urgent care, an emergency department (ED) visit, or hospitalization for any cause.

### Data Retrieval

Demographic data and medical history were collected via participant interview, chart reviews, and clinical databases. These data were stored in a REDCap database (www.projectred cap.org) hosted at the University of Calgary Clinical Research Unit. A Fitbit account was created for each participant using the Fitbit dashboard (fitbit.com) and was registered using a unique email address generated by the University of Calgary’s information technology department. The research assistant met with each patient weekly during regular hemodialysis treatments to sync the Fitbit to the dashboard and download data from the tracker. Any missed dialysis treatments in the previous week were recorded. Participants were asked about any period since the last study visit during which the Fitbit tracker was not worn.

All participant urgent care visits, ED visits, and hospitalizations between June 29, 2019, and November 14, 2019 (during the period that the Fitbit trackers were worn), were extracted from the National Ambulatory Care Reporting System (NACRS) and the Discharge Abstract Database (DAD) from within the Alberta Health Services Enterprise Data Warehouse, with support provided from the Alberta SPOR Support Unit Data Platform. The daily ambient temperature in Calgary was retrieved from the Weather Network (www.theweathernetwork.com).

### Analyses

We did analyses with Stata MP 15·1 (www.stata.com) and reported baseline descriptive statistics as counts and percentages, or medians and interquartile limits, as appropriate. Box and whisker plots and line plots were used to describe each participant’s daily steps. Adjusted rate ratios (and 95 % confidence intervals) were calculated to measure the associations between the number of steps taken each day with a number of potential predictors: age, sex, body mass index (BMI) (categorized as <18.5, 18.5-25, 26-29, and ≥30 kg/m^2^), primary cause of end-stage kidney disease (diabetic nephropathy, hypertensive nephropathy, glomerulonephritis, vascular nephropathy, polycystic kidney disease, and other), dialysis vintage (2018-2019, 2015-2017, <2015), whether the participant had dialysis that day or not, whether the participant had an arteriovenous fistula or catheter, whether the participant was enrolled in the in-center dialysis biking program, the performance index, and the daily ambient temperature. Frailty score was not considered as a potential predictor because it was highly correlated with the performance index (*r* = −0.81). We used mixed negative binomial regression with a natural logarithm link to estimate these associations because negative binomial regression, unlike Poisson regression, allows for overdispersion. Because participants had many days of follow-up observation, we used a random effect for participant. We considered a fully adjusted model and an adjusted model built using backward elimination. The threshold *P* for statistical significance was set at 0.05.

In a sensitivity analysis, we considered the product of body mass in kilograms multiplied by the daily total number of steps (“work”). We wanted to see if the association between BMI and work was different than the association between BMI and steps. In an additional sensitivity analysis, we evaluated the number of steps using 3-day moving averages (calculated as the mean of the previous 3 days), reasoning that this might be more stable within individuals than the mean number of steps.

### Sample Size

We aimed to evaluate a convenience sample of 60 participants. Because this was a feasibility study, a formal power calculation was not done.

## Results

### Characteristics of Participants

We screened 98 hemodialysis patients for inclusion in the study; 6 were ineligible, and 45 declined to participate (Fig S1). Forty-seven patients were enrolled. Six participants stopped wearing the Fitbit trackers before the end of the follow-up period. Of those, 1 did not contribute any step data and was excluded from the analyses, thus 46 participants were included in the analysis. Study flow is shown in Figure S1. The dataset was locked on March 12, 2020.

The median age of the participants was 64 years (range, 22 to 85 years), and 63 % were men (Table 1). The median BMI was 26 kg/m^2^ (range, 19 to 46 kg/m^2^). The most frequent cause of kidney failure was vascular nephropathy (with 30 %). Seventy percent of participants had a hemodialysis catheter; most (87 %) had initiated hemodialysis in the last 4 years. Fifty-nine percent were enrolled in the unit’s intradialytic biking program. The median frailty score was 4, and the median performance index was 70; both indicate a vulnerable person who is not dependent on others but has his or her activities limited due to symptoms. The frailty score range was 2 to 7, from generally well (someone with no active disease symptoms) to severely frail.

**Table 1:** Demographics and Clinical Characteristics

Characteristic	N (%) or Median [IQR] (Range)
N	46
Age, y	64 [49-71] (22-85)
Female	17 (37.0 %)
BMI, kg/m^2^	26 [22-28] (19-46)
Cause of kidney failure	
Diabetic nephropathy	9 (19.6 %)
Hypertensive nephropathy	8 (17.4 %)
Glomerulonephritis	6 (13.0 %)
Vascular nephropathy	14 (30.4 %)
PCKD	4 (8.7 %)
Other	5 (10.9 %)
Dialysis vintage	
2018-2019	21 (45.7 %)
2015-2017	19 (41.3 %)
<2015	6 (13.0 %)
CVC	32 (69.6 %)
AVF	15 (32.6 %)
Diabetes	17 (37.0 %)
Sleep apnea	2 (4.3 %)
Enrolled in biking program	27 (58.7 %)
Performance index	70 [60-80] (40-90)
Clinical frailty score	4 [3-5] (2-7)

Abbreviations: AVF, arteriovenous fistula; BMI, body mass index; CVC, central venous catheter; IQR, interquartile range; PCKD, polycystic kidney disease.

### Total Daily Steps

**Figure 1:**
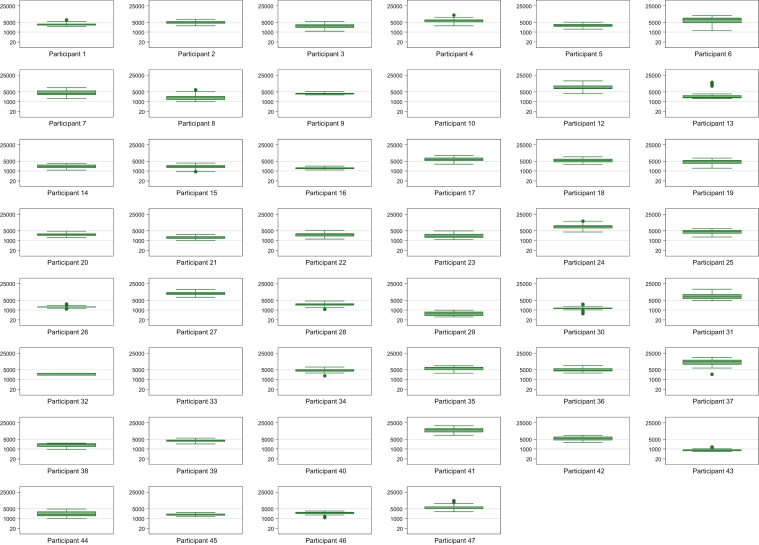
Distribution of daily steps for each individual participant. The box and whisker plots are shown on a cube root scale, but the axis is labeled with the natural scale.

Participants accumulated a median of 31 days of step data (interquartile range [IQR], 27-31; range, 2-43). The median number of steps taken per day was 3,133 (IQR, 1,976-5,097; range, 248-13,753). Figure 1 shows the distribution of daily steps taken by each participant; figure S2 shows the distribution of daily steps taken by all participants combined. The mean daily ambient temperature in Calgary ranged from −10.7 to 31.4°C (median 17.8°C).

**Figure 2:**
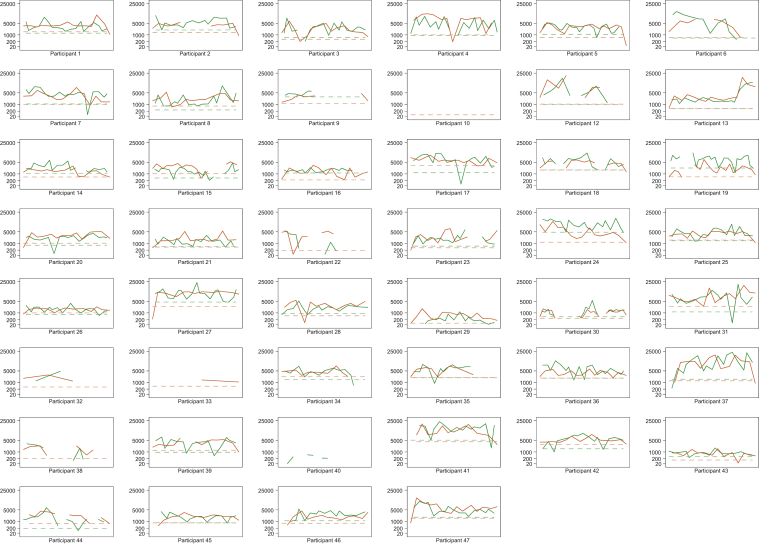
Total steps by days of follow-up for each individual participant: dialysis and nondialysis days. The *y*-axis of the line plots are shown on a cube root scale, but the *y*-axis is labeled with the natural scale. The *x*-axis shows the days that a Fitbit was worn by each individual participant. The red line connects the total steps per each dialysis day. The green line connects the total steps per each nondialysis day. The dotted lines show the individual participant’s lower 80 % confidence limit for dialysis (red) and nondialysis (green) days.

Table 2 shows the associations between clinical characteristics and the number of steps taken each day. Step count was not significantly different between the dialysis and nondialysis days (fig 2); after dropping the not statistically significant covariates, only age, BMI, performance index, and ambient temperature were significantly associated with the daily number of steps. Better performance and higher ambient temperature were associated with more daily steps (1.01 per unit; 95 % CI, 1.00-1.03; and 1.01 per °C; 95 % CI, 1.00-1.01). Increasing age and obesity (BMI ≥ 30 kg/m^2^) were associated with a fewer number of daily steps (0.98 per year; 95 % CI, 0.97-0.99; and 0.63; 95 % CI, 0.42-0.96).

**Table 2:** Associations With Total Steps and Work per Day

Covariate	Daily Steps, RR (95 % CI)		Daily Work, RR (95 % CI)		3-Day Moving Mean, RR (95 % CI)	
	Fully Adjusted	Adjusted	Fully Adjusted	Adjusted	Fully Adjusted	Adjusted
N/days	46/1,275	46/1,275	46/1,275	46/1,275	43/1,119	43/1,119
Age, y	0.98 (0.97-0.99)^a^	0.98 (0.97-0.99)^a^	0.98 (0.97-0.99)^a^	0.98 (0.97-0.99)^a^	0.98 (0.97-0.99)^a^	0.98 (0.97-0.99)^a^
Female	0.87 (0.63-1.19)	—	0.75 (0.54-1.03)	—	0.79 (0.60-1.06)	—
BMI, kg/m^2^						
18.5-25	1.00	1.00	1.00	1.00	1.00	1.00
26-29	1.37 (0.96-1.97)	1.26 (0.86-1.84)	1.70 (1.19-2.42)^a^	1.57 (1.06-2.32)^a^	1.23 (0.90-1.69)	1.17 (0.84-1.64)
≥30	0.79 (0.52-1.19)	0.63 (0.42-0.96)^a^	1.30 (0.86-1.96)	0.96 (0.63-1.47)	0.77 (0.52-1.14)	0.61 (0.42-0.89)^a^
Cause of kidney failure						
Diabetic nephropathy	1.00	—	1.00	—	1.00	—
Hypertensive nephropathy	0.90 (0.53-1.53)	—	1.17 (0.70-1.98)	—	0.90 (0.56-1.45)	—
Glomerulonephritis	1.34 (0.78-2.28)	—	1.59 (0.94-2.71)	—	1.15 (0.72-1.85)	—
Vascular nephropathy	1.08 (0.69-1.69)	—	1.40 (0.90-2.18)	—	1.28 (0.86-1.91)	—
PCKD	1.22 (0.68-2.21)	—	1.33 (0.74-2.39)	—	1.22 (0.73-2.05)	—
Other	1.60 (0.91-2.81)	—	1.94 (1.11-3.39)^a^	—	1.53 (0.93-2.50)	—
Dialysis vintage						
2018-2019	1.00	—	1.00		1.00	
2015-2017	0.75 (0.52-1.09)	—	0.79 (0.55-1.15)	—	0.84 (0.60-1.19)	—
<2015	1.15 (0.70-1.91)	—	1.27 (0.77-2.10)	—	1.09 (0.69-1.72)	—
Dialysis day	0.96 (0.89-1.03)	—	0.96 (0.90-1.03)	—	0.99 (0.96-1.04)	—
AVF	1.38 (0.99-1.93)	—	1.39 (1.00-1.94)^a^	—	1.38 (1.01-1.90)^a^	—
Enrolled in biking program	1.01 (0.73-1.39)	—	1.02 (0.75-1.41)	—	1.07 (0.80-1.42)	—
Performance index	1.01 (1.00-1.03)^a^	1.01 (1.00-1.03)^a^	1.01 (1.00-1.02)	1.01 (1.00-1.02)^a^	1.02 (1.01-1.03)^a^	1.02 (1.01-1.03)^a^
Ambient temp, °C	1.01 (1.00-1.01)^a^	1.01 (1.00-1.01)^a^	1.01 (1.00-1.01)^a^	1.01 (1.00-1.01)^a^	1.01 (1.00-1.01)^a^	1.01 (1.00-1.01)^a^

Work is defined as a participant’s weight in kilograms multiplied by the number of daily steps. The full models adjusted for age, sex, BMI (categorized as <18.5, 18.5-25, 26-29, and ≥30 kg/m^2^), primary cause of end-stage kidney disease (diabetic nephropathy, hypertensive nephropathy, glomerulonephritis, vascular nephropathy, PCKD, and other), dialysis vintage (2018-2019, 2015-2017, <2015), whether the participant had dialysis that day or not, whether the participant had an AVF or catheter, whether the participant was enrolled in the in-center dialysis biking program, the performance index, and daily ambient temperature. The covariates for the adjusted models were selected using backward elimination with *P* < 0.05.

Abbreviations: AVF, arteriovenous fistula; BMI, body mass index; CI, confidence interval; CVC, central venous catheter; IQR, interquartile range; PCKD, polycystic kidney disease; RR, rate ratio.

a Statistically significant associations.

In the sensitivity analysis, when work was regressed on the potential predictors, obesity was not associated with less work compared with the participants with BMIs between 18.5 and 25.0 kg/m^2^. However, overweight status (BMI between 26 and 29 kg/m^2^) was associated with more work than for participants with BMIs between 18.5 and 25.0 kg/m^2^. The model using the 3-day moving average gave similar results (Table 2; Figs S3 and S4).

Few events were reported (Table 3). Of the 46 patients, 11 reported at least 1 event for a total of 14 events. There were 4 urgent care visits (among 3 patients), 7 ED visits (among 6 patients), and 3 hospitalizations (among 3 patients). There was no clear relationship between the clinical events and the within-patient pattern in the number of daily steps (or the 3-day moving average of steps).

**Table 3:** Events During Study Period

Record ID	Event	Date	Primary Diagnosis	
2	ED visit	Day 9 of 29	T82.8	Other specified complications of cardiac and vascular prosthetic devices, implants and grafts
2	ED visit	Day 25 of 29	J18.9	Pneumonia, unspecified
5	Urgent care	Day 20 of 36	T79.3	Post-traumatic wound infection, not elsewhere classified
5	Urgent care	Day 33 of 36	L02.4	Cutaneous abscess, furuncle and carbuncle of limb
6	ED visit	Day 16 of 20	R94.31	Abnormal cardiovascular function studies (biomarkers or ECG) suggestive of NSTEMI
6	Hospitalization	Days 17-19 of 20	I21.4	Acute subendocardial myocardial infarction
10	ED visit	2 Sep 2019 Day 15 of 15	S06.0	Concussion
15	Hospitalization	Days 33,34 of 42	T82.8	Other specified complications of cardiac and vascular prosthetic devices, implants and grafts
20	Urgent care	Day 20 of 32	N48.1	Balanoposthitis
21	Urgent care	Day 24 of 32	J06.9	Acute upper respiratory infection, unspecified
22	ED visit	Day 17 of 29	R10.4	Other and unspecified abdominal pain
26	ED visit	Day 23 of 34	S60.9	Unspecified superficial injury of wrist and hand
37	Hospitalization	Day 3 of 30	Z49.0	Preparatory care for dialysis
40	ED visit	Day 10 of 16	S20.2	Contusion of thorax

Abbreviations: ECG, electrocardiogram; ED, emergency department; NSTEMI, non-ST segment elevation myocardial infraction.

## Discussion

In our study of 46 hemodialysis patients, we found a median number of steps taken per day of 3,133, with interquartile range of 1,976 to 5,097 steps. The median age of participants was 64 years, and 63 % were men. Increasing age and the presence of obesity were associated with a fewer number of daily steps. A higher daily ambient temperature was associated with more daily steps. Baseline performance index and vascular access type were also associated with daily step count; better performance and having an arteriovenous fistula (as opposed to a central venous catheter) were associated with more daily steps. There was no association between step count and the occurrence of clinical events, although the number of events was very small.

Our study aimed to identify patient-specific step counts that could be used to identify days with lower-than-usual activity, which in turn could be a proxy for subclinical illness. Therefore, the wide observed between-participant variation in activity level was not problematic because the aim was to identify a within-patient normal range. However, we were unable to identify individual-specific normal ranges because of substantial within-patient variation in daily step counts. Step count varied within individual patients over the 4-week study but somewhat surprisingly was not consistently affected by the timing of dialysis treatment. The overall number of steps taken was relatively low on most days, perhaps reflecting the burden of age and comorbidity among contemporary hemodialysis patients. Although we did find that patients tended to walk more on warmer days, weather alone may not explain the variation.

A possible explanation of the high within-patient variability may be related to the accuracy of wearable fitness trackers. Multiple studies have shown that Fitbit trackers have high error rates,^6^, ^7^, ^8^, ^9^, ^10^, ^11^ particularly when estimating step count among slow-walking populations.^7^ This may be related to the accelerometer on the wristband^10^ and its limited ability to track motion in some people who do not move their arms when walking. However, this would likely affect between-patient variation more than within-patient variability, and even among smartphone trackers that do not rely on a wristband accelerometer, step count accuracy is relatively poor.^12^^,^^13^ Further, wearable tracker technology is constantly evolving and is not consistent across different products,^6^, ^7^, ^8^, ^9^^,^^11^ We speculate that future products may be more accurate and thus may reduce the observed within-patient variability in step count.

Other reasons that may account for the high within-patient step count variability could be simply that people do not have consistent walking patterns over time. Our study identified that weather influenced step count, and it is likely that other factors, such as work or personal schedules and mood may change an individual’s daily activity level. Further, the expected fluctuations in energy level associated with treatment of a chronic illness such as kidney failure may also have contributed to the observed within-patient variability in activity. Although using a moving average of step count over 3 days did reduce the within-patient variability as compared with mean daily step count, considerable variability remained. Therefore, future studies could explore the within-patient variability and predictive power associated with moving averages for daily step counts over a longer period (eg, 5, 7, or 9 days), ideally in a larger patient sample and with a longer duration of follow-up.

Previous studies in people with diabetes or with a kidney transplant have suggested that activity trackers may help to motivate people to increase their physical activity,^14^^,^^15^ but whether they can be used to predict clinical deterioration in people with chronic illness is uncertain. Other studies exploring hemodialysis patients’ walking patterns have reported varying daily step count, ranging from a median of 3,688^16^ to 6,393^17^ steps per day. There are a number of potential reasons for these differences; however, all reported wide between-participant variation in the number of daily steps.

Hemoglobin level, lower extremity muscle strength, and physical function were found to correlate significantly with walking time and active time among hemodialysis patients^18^ and may account for this variability. Patient phenotypes such as inflammation, cardiovascular disease, protein-energy wasting, and diabetes have also been shown to reduce walking levels among dialysis patients.^16^ Other factors not related to patient health have been found to influence walking patterns of hemodialysis patients: for example, the day of the week (some patients tend to walk less on a Sunday^19^) or neighborhood walkability.^17^

Our study has certain limitations that should be considered when interpreting results. First, technologies or activities that promote health through behavior change may be at particular risk of the novelty effect, and it is possible that transient increases in activity associated with receiving the tracker or the Hawthorne effect may have increased within-patient variability during the study period. Following patients for a longer duration (eg, 6 months or 1 year) or excluding data collected during an initial run-in period might have helped to address this issue. Second, it is possible that some participants did not consistently wear their Fitbit trackers, which may have led to measurement error and spuriously increased apparent within-patient variability in activity. However, because this limitation would likely occur in real-world use, it does not necessarily threaten our conclusion that activity trackers may not be useful for predicting clinical deterioration.

Third, the number of participants was relatively small, and there were too few events to model to examine whether step count could predict clinical deterioration. However, although step count metrics were not sufficient to develop a robust prediction algorithm, future research exploring the accuracy and predictive potential of other wearable technology metrics (ie, heart rate, active minutes, sleep time and quality, etc) as the technologies advance may be useful to help answer this question.

Fourth, to reduce the impact of within-patient variability in activity from day to day, it might have been preferable to identify patient-specific norms over a longer period, such as the mean number of steps over 3-day or even 7-day periods. Our analyses suggested that there was still significant within-patient variability in these parameters, although this hypothesis could be tested in future studies using a longer period of data collection and perhaps using multivariate models to predict patient-specific norms.

Finally, our study population was drawn from a single hemodialysis program in Alberta, Canada, and not all eligible participants agreed to participate, which may have introduced selection bias. For example, nearly 70 % of the study population was treated with a central venous catheter rather than with arteriovenous access. Therefore, whether our results apply to all people receiving maintenance hemodialysis or other clinical populations would require further study.

In conclusion, the median step count among hemodialysis patients was 3,133, with a wide range between participants of 248 to 13,753. Ambient temperature and certain patient-related factors were significantly associated with mean step count, but we observed substantial within-patient variation during the study. Consequently, it was not possible to identify patient-specific normal ranges, which in turn makes it less likely that activity trackers will be useful for predicting clinical deterioration, at least in the population studied.
