# Supplementary material for: Wearable Fitness Trackers to Predict Clinical Deterioration in Maintenance Hemodialysis: A Prospective Cohort Feasibility Study
Source: Kidney Med. 2021 Jun 29;3(5):768–775.e1. doi: 10.1016/j.xkme.2021.04.013 (PMC8515069; doi:10.1016/j.xkme.2021.04.013)
Supplement: Supplementary File (PDF) — Figures S1-S4. [file mmc1.pdf]

Figure S1. Participant flow

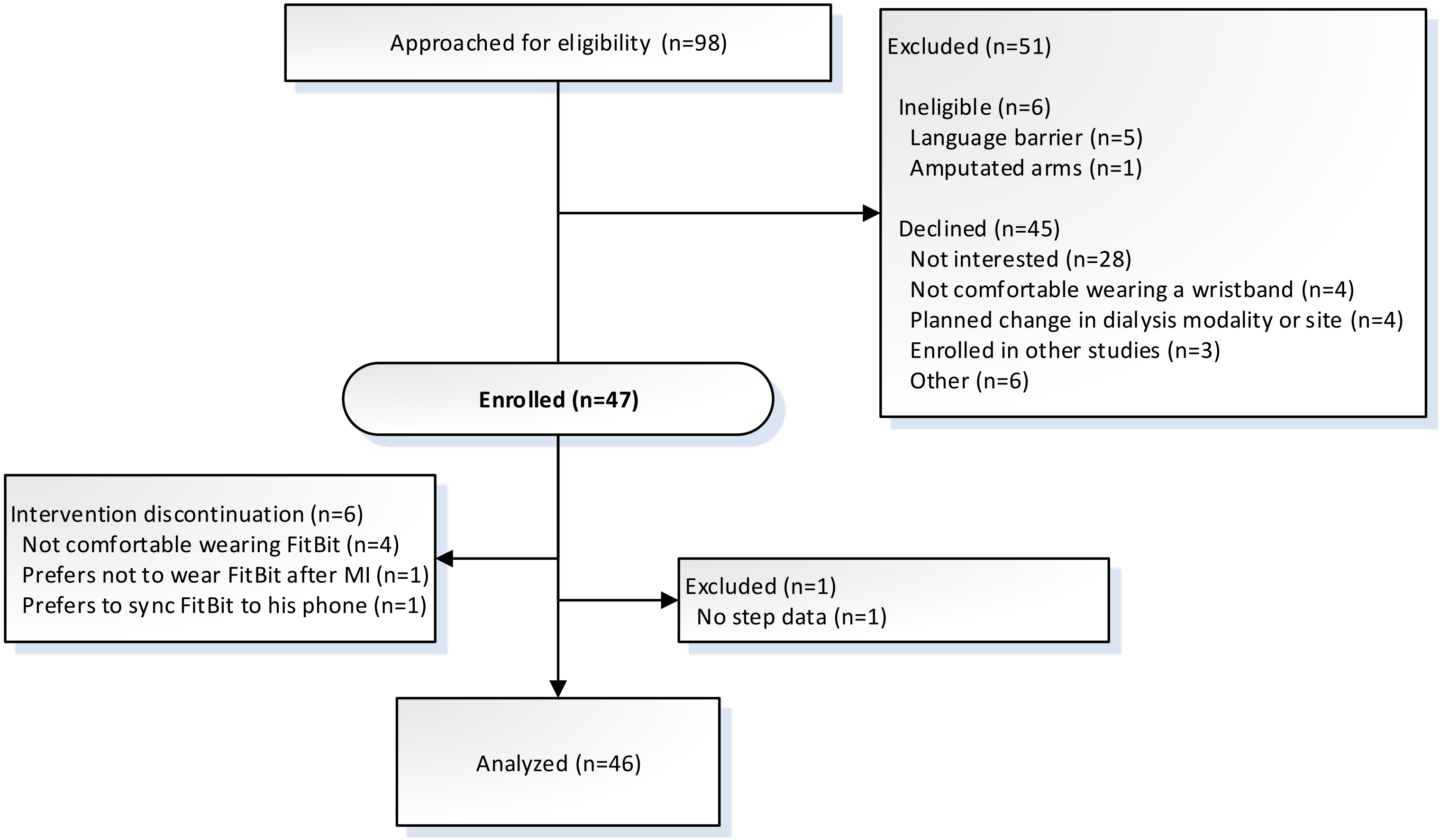

Figure S2. Box plots for all participants: daily step count (left) and 3-day moving average (right).

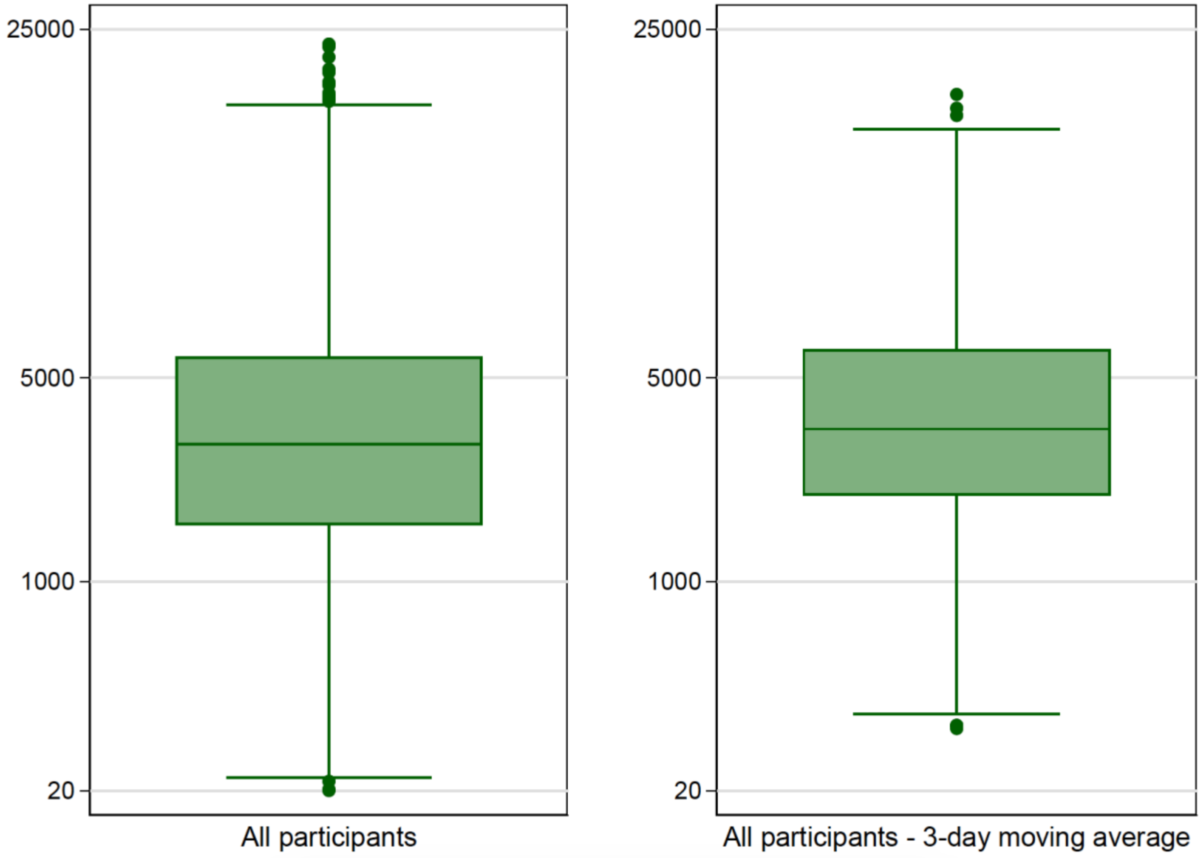

Figure S3. Distribution of 3-day moving average step count for each individual participant

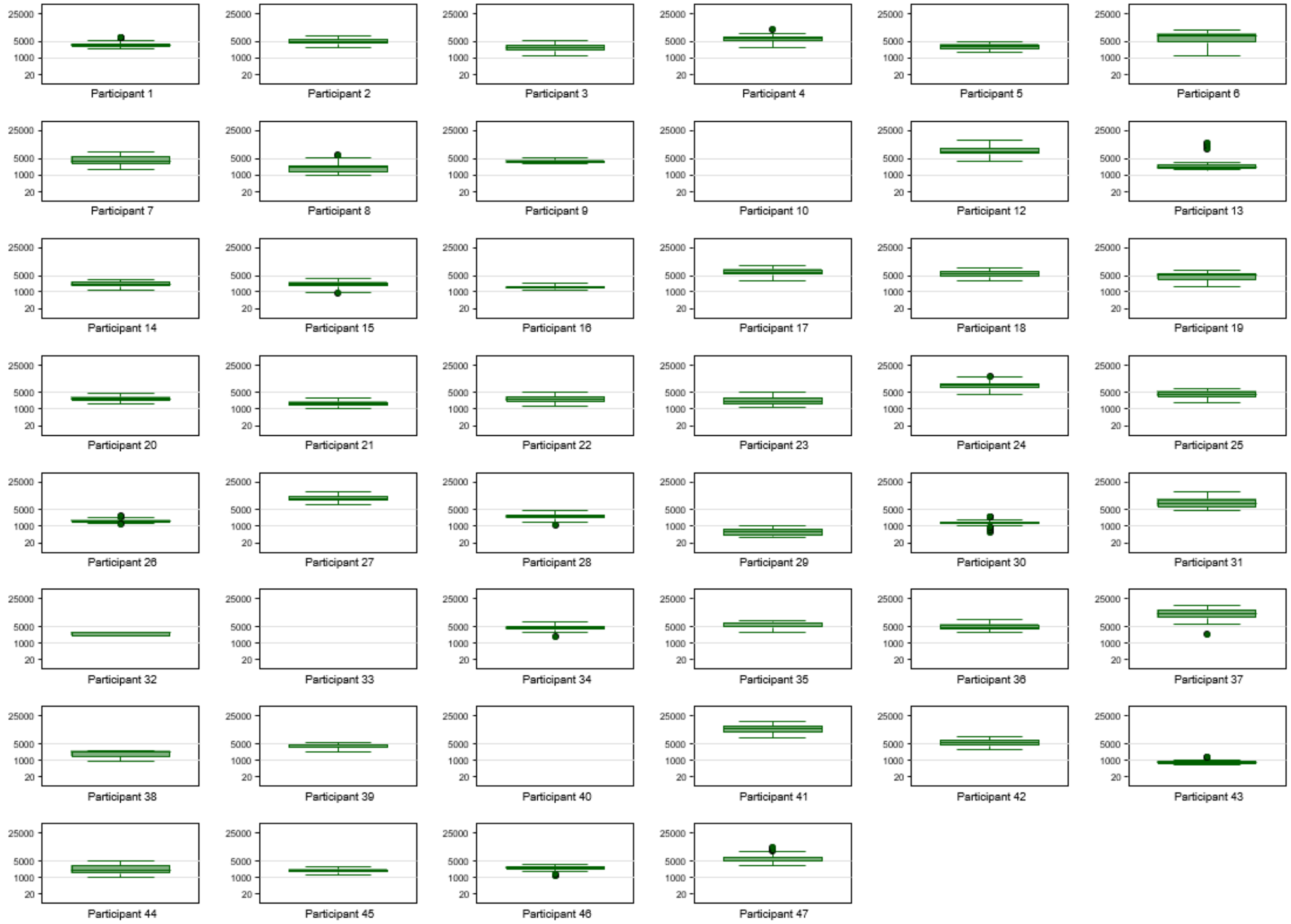

The box and whisker plots are shown on a cube root scale however the axis is labelled with the natural scale.

Figure S4. 3-day moving average step count by days of follow-up for each individual participant

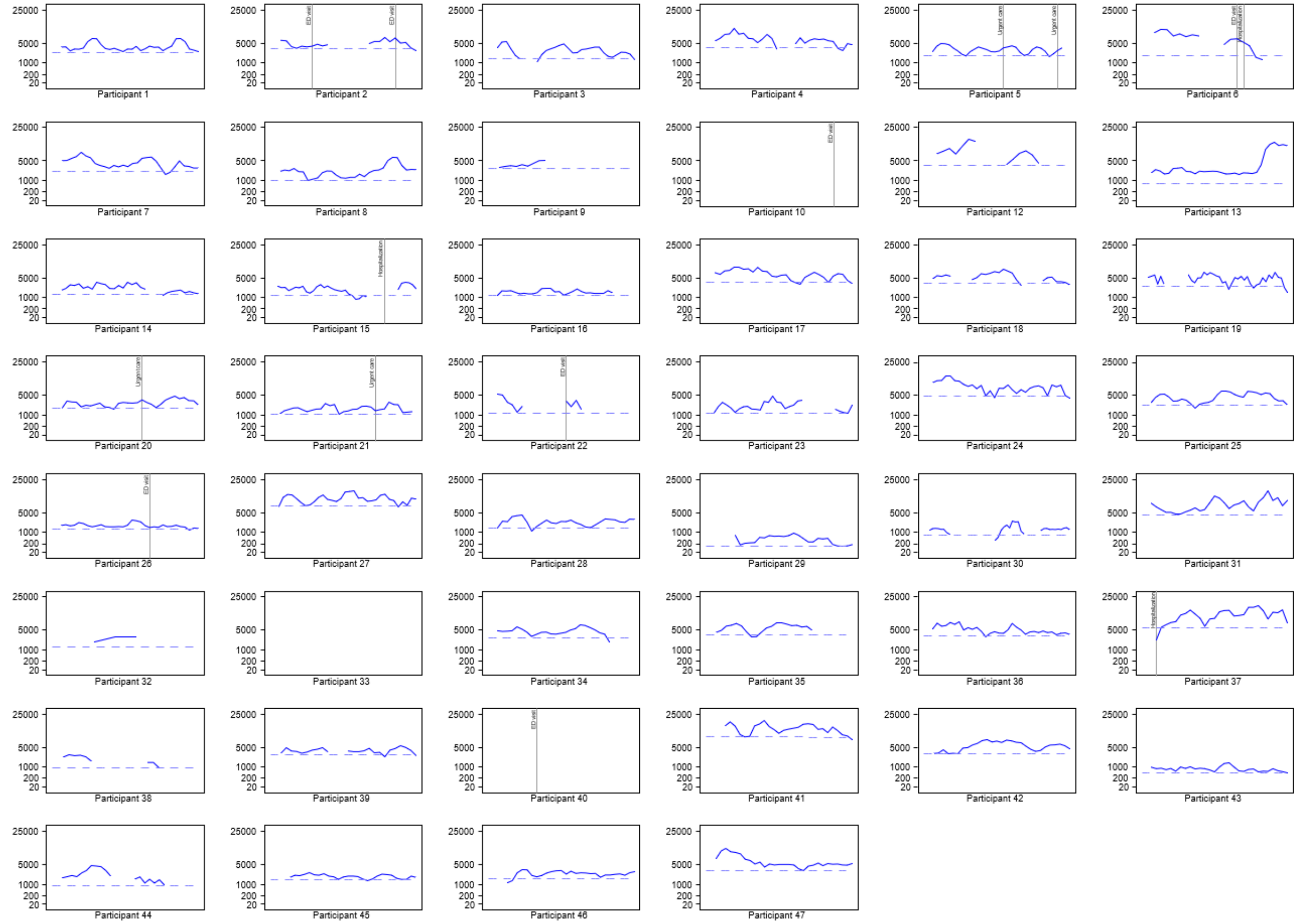

The y-axis of the line plots are shown on a cube root scale however the y-axis is labelled with the natural scale. The x-axis shows the days that a Fitbit was worn by each individual participant. The blue line connects the 3-day moving average step count; the timing of dialysis treatments was not considered. The dotted line shows the individual participant’s lower 80% confidence limit.
